# Removal of the Placenta

**Published:** 1861-07

**Authors:** 


Foreign Items.—We condense the following extracts from Dr. Elsberg's *Summary of Foreign Medical News* in the *Am. Medical Monthly* for June,—a most valuable and interesting department in that excellent periodical. In an article by Dr. Crede, on the most efficient mode of removing the placenta, it is strongly insisted that the removal shall be the result of uterine action alone, restricting the introduction of the hand in the genitalia to exceptional, extremely rare and urgent cases. His method consists in excitation and increase of uterine activity by friction and irritation of the fundus and body of the uterus with the hand through the abdominal covering. This manipulation must be performed at once after the birth of the child. At first the friction must be gentle; gradually we may increase it; the author says he has in innumerable cases, without a single exception, succeeded in a quarter or half hour in inducing the necessary artificial contraction, even where there was previously ever so slight uterine action. When the contraction attains its maximum force, he grasps the whole uterus in such a manner that the fundus lies in the hollow of his hand, and the five fingers around the body may still exert a gentle pressure. He says he felt under his fingers the placenta leave the womb in every case; and gen-erally this occurred with such a degree of force, that it at once protruded from the external genitalia, or at least was found lying in the lower part of the vagina. The patient suffers no other inconvenience than the increased pain accompanying the more forcible contraction, which, however, is more than compensated for, by its rendering unnecessary the introduction of the finger or hand in the parts already sore and morbidly sensitive by the previous tension and traction during labor. The womb afterwards remains well contracted; post-partum hæmorrhage is not so likely to occur; inversion can never take place with a regular contraction; while, with the usual proceeding for taking away the after-birth, the greatest care does not preclude the possibility of its occurrence.

